# Challenges in Saving Trauma Patients in Seoul Based on the 2016–2020 Community-Based Severe Trauma Survey

**DOI:** 10.3390/jcm14051471

**Published:** 2025-02-22

**Authors:** Hoonsung Park, Seung Min Baik, Hangjoo Cho, Maru Kim, Jae-Myeong Lee

**Affiliations:** 1 Division of Acute Care Surgery, Department of Surgery, Korea University Anam Hospital, Seoul 02841, Republic of Korea; niedwynn@naver.com; 2 Division of Critical Care Medicine, Department of Surgery, Ewha Womans University Mokdong Hospital, Ewha Womans University College of Medicine, Seoul 07985, Republic of Korea; illaillailla@naver.com; 3 Department of Trauma Surgery, Uijeongbu St. Mary’s Hospital, College of Medicine, The Catholic University of Korea, Seoul 06591, Republic of Korea; surgeryman@catholic.ac.kr (H.C.); maru@catholic.ac.kr (M.K.)

**Keywords:** Seoul, Republic of Korea, multiple trauma, risk factors, mortality

## Abstract

**Background/Objectives**: The preventable trauma death rate (PTDR) reflects the quality of trauma management systems. In the Republic of Korea, the PTDR in Seoul, the capital city, decreased from 30.8% in 2015 to 20.4% in 2019. However, it remains the highest in the country. In contrast, Gyeonggi-Incheon, which includes Gyeonggi Province and Incheon Metropolitan City surrounding Seoul, had the lowest nationwide PTDR (27.4% in 2015 to 13.1% in 2019). This study aimed to investigate the characteristics and in-hospital mortality risk factors for patients with trauma in Seoul and Gyeonggi-Incheon. **Methods**: This retrospective cohort study used data from a 2016 to 2020 Community-Based Severe Trauma Survey. Among 237,616 patients, 24,448 were included in the study after applying the inclusion and exclusion criteria. **Results**: The proportions of the population with motor vehicle and workers’ compensation insurance increased with increasing injury severity in both regions. The injury severity score (ISS) was significantly higher in Gyeonggi-Incheon in the ISS < 9 and ISS 9–15 groups. Across all hospital levels, the proportion of patients who visited regional trauma centers in Seoul was low across all three ISS groups (0.2%[*n* = 26], 0.6%[*n* = 23], and 1.9%[*n* = 56] for ISS < 9, ISS 9–15, and ISS > 15, respectively). Conversely, in Gyeonggi-Incheon, the proportion of patients who visited regional trauma centers increased as injury severity increased across all three ISS groups (37.3%[*n* = 1404], 50.6%[*n* = 732], and 64.4%[*n* = 856] for ISS < 9, ISS 9–15, and ISS > 15, respectively). In Seoul, the identified in-hospital mortality risk factors included age, National Health Insurance (NHI) loss, other insurance, ISS, regional and local emergency centers or institutes, and the number of angioembolizations. In Gyeonggi-Incheon, the in-hospital mortality risk factors included age, ISS, falls and slippage, and the number of angioembolizations. **Conclusions**: The unique in-hospital mortality risk factors in Seoul compared with those in Gyeonggi-Incheon include transfers to regional emergency centers (ISS > 15), local emergency centers or institutes (ISS > 15), NHI loss (ISS 9–15 and ISS > 15), and the use of other insurance (ISS > 15).

## 1. Introduction

The establishment of the trauma system in the Republic of Korea was marked by the designation of regional trauma centers in 2012 and the opening of the Incheon, Chungnam, and Jeonnam regional trauma centers in 2014. Seventeen centers were designated, and the last center was established by December 2023. Regional trauma centers in the Republic of Korea are level 1 trauma centers [1].

The preventable trauma death rate (PTDR) is defined as the death rate considering the percentage of patients who could have survived if they had been transported to an appropriate hospital within an appropriate period and provided appropriate medical treatment. In the Republic of Korea, preventable trauma deaths were evaluated at each trauma center through a multidisciplinary panel review in accordance with the World Health Organization guidelines for trauma quality improvement programs. The panel chair leads discussions to categorize each case as (1) preventable, (2) potentially preventable, (3) non-preventable, or (4) non-preventable with inadequate care [2]. Table S1 [3] outlines the definitions for each category. The appropriateness of transportation, time, and medical treatment is determined by a panel review based on definitions of preventability.

The PTDR reflects the quality of the trauma system [4,5]. In the United States, a collaborative effort has been initiated between military and civilian trauma systems to achieve zero preventable deaths [6]. In the Republic of Korea, the PTDR decreased from 30.5% in 2015 to 15.7% in 2019 [7]. The PTDR in Seoul decreased from 30.8% in 2015 to 20.4% in 2019. However, it remains the highest in the country. In contrast, the Gyeonggi-Incheon region had the lowest PTDR nationwide, decreasing from 27.4% in 2015 to 13.1% in 2019 [8].

The trauma centers in the United States are classified as Level 1, 2, and 3. Level 1 trauma centers must be capable of providing system leadership and comprehensive trauma care for all types of injuries. In this central role, a Level 1 trauma center must have sufficient resources and personnel. Most Level 1 trauma centers are university-based teaching hospitals, due to the extensive resources required for patient care, education, and research. Level 2 trauma centers are expected to provide initial definitive trauma care for a wide range of injuries and severities. Additionally, they may take on regional responsibilities related to education, system leadership, and disaster planning. Level 3 trauma centers typically serve communities in more remote and/or rural areas that may not have timely access to a Level 1 or 2 trauma center [9].

In the Republic of Korea, regional trauma centers function as Level 1 trauma centers. However, there are no designated lower-level trauma centers, such as Level 2 or 3. Instead, trauma patients are managed at either regional emergency centers or local emergency centers/institutes [10]. Seoul, the capital city of the Republic of Korea, established four Final Treatment Centers for Severe Trauma, with hospitals striving to function as Level 2 trauma centers—except for one center, which is duplicately designated as both a regional trauma center and a Final Treatment Center for Severe Trauma.

This study aimed to investigate the characteristics of trauma patients in the Republic of Korea and the risk factors for in-hospital mortality, focusing on Seoul, which has the highest PTDR, and the Gyeonggi-Incheon region, which has the lowest PTDR.

## 2. Materials and Methods

### 2.1. Study Design and Data Collection

This retrospective cohort study used data from a 2016 to 2020 Community-Based Severe Trauma Survey conducted by the Korea Disease Control and Prevention Agency. This database contains data from the National Fire Agency and 119 Rescue and medical records obtained from hospitals between 2016 and 2020. They were de-identified according to the Personal Information Protection and Statistics Act. Trained investigators visited hospitals identified by 119 Rescue transport records. They collected data from the medical records to verify whether the patients met the inclusion criteria in the database. A transfer survey was conducted to verify additional treatments and outcomes of patients transferred from primary hospitals to other hospitals.

The database, although not precisely the same as the registry, included patients transferred to the hospital by 119 Rescue services, Korean emergency services, trauma, non-traumatic injuries, and multiple injury cases. According to the definition in the registry, patients of all ages, with trauma, and of both sexes, were involved in injury mechanisms, including motor vehicle collisions, falls and slippage, blunt injuries, penetrative injuries, and machinery-related incidents. Non-traumatic injuries include thermal injuries, hanging, airway obstruction due to external objects, and drowning. Multiple injuries include those from natural disasters, accidents involving public transportation, exposure to chemical, biological, or radiological substances, and injuries during mass gatherings.

### 2.2. Study Population

**Figure 1:**
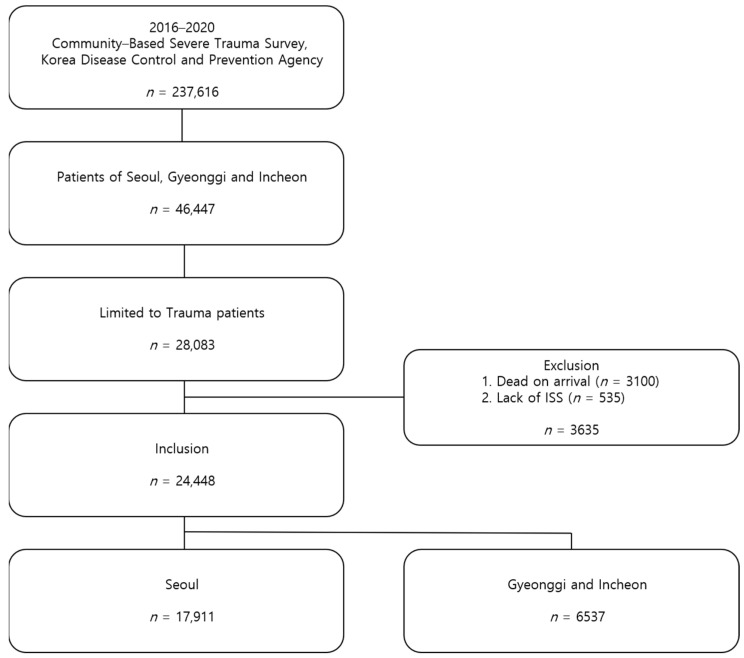
Flow diagram of the study population. ISS, Injury Severity Score.

Among the entire database of 237,616 patients, 46,447 patients were from the Seoul and Gyeonggi-Incheon regions. In this subset, 28,083 patients had experienced trauma. In this database, trauma was defined as injuries caused by motor vehicle collisions, falls, slippage, blunt injuries, penetrative injuries, or machinery-related incidents. Patients who died on arrival (3100) and 535 with missing injury severity scores (ISS) were excluded. A total of 24,448 patients (17,911 from Seoul and 6537 from Gyeonggi Province and Incheon Metropolitan City) were included in this study (Figure 1).

### 2.3. Study Settings

#### Emergency Medical Facility Type in the Republic of Korea

**Figure 2:**
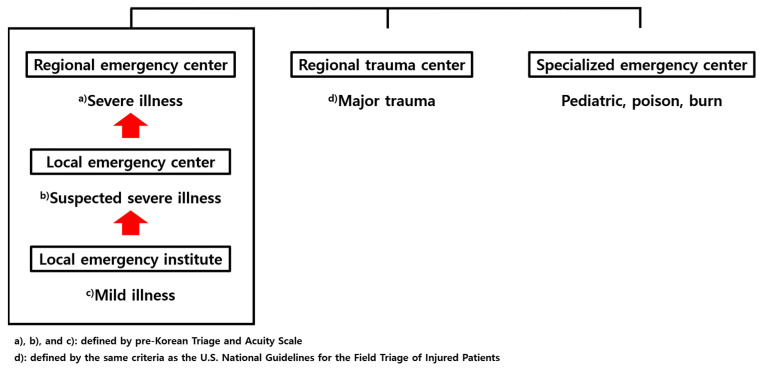
Emergency medical facilities in the Republic of Korea.

In the Republic of Korea, emergency medical facilities are categorized into regional emergency centers, local emergency centers, local emergency institutes, specialized emergency centers, and regional trauma centers. This sequential emergency medical delivery system conceptually prioritizes patients with severe emergencies for treatment at regional centers. In contrast, patients with mild or suspected severe emergencies are treated at local emergency centers and institutes (Figure 2).

Field triage is used to assess the severity of emergencies. In the Republic of Korea, field triage utilizes the pre-Korean Triage and Acuity Scale (pre-KTAS). This system, which was piloted in 2022 and implemented nationwide in 2024, classifies patients into five levels based on their initial impression, illness, and symptoms: Level 1 and 2 (severe), Level 3 (suspected severe), and Level 4 and 5 (mild). Typically, Levels 1 and 2 are recommended for transport to a regional emergency center, Levels 2 and 3 to a local emergency center, and Levels 4 and 5 to a local emergency institute [11].

Prior to 2022, which corresponds to the timeframe of this database, field triage was conducted using the 119 Rescue triage system rather than pre-KTAS. Under this system, patients with major trauma were recommended for transport to the nearest regional trauma center, while those with non-major trauma were advised to be transported to the nearest local emergency institute. The definition of major trauma follows the same criteria as the U.S. National Guidelines for the Field Triage of Injured Patients [12]. Specialized emergency centers are available for pediatric, poisoned, and burnt patients. Regional trauma centers are dedicated to major trauma cases [13].

Recognizing the critical need for an efficient trauma care system, the Korean government organized the nation into five major regions based on resource adequacy, population demand, and accessibility. By 2017, it had designated 17 regional trauma centers according to national standards, including those established by the Ministry of Health and Welfare (MOHW) [14]. However, still many involved in 119 Rescue and members of the public are confused with the concept, and indications for the use of different centers thus transfer between regional emergency centers and regional trauma centers.

### 2.4. Trauma Center Status in Seoul

**Figure 3:**
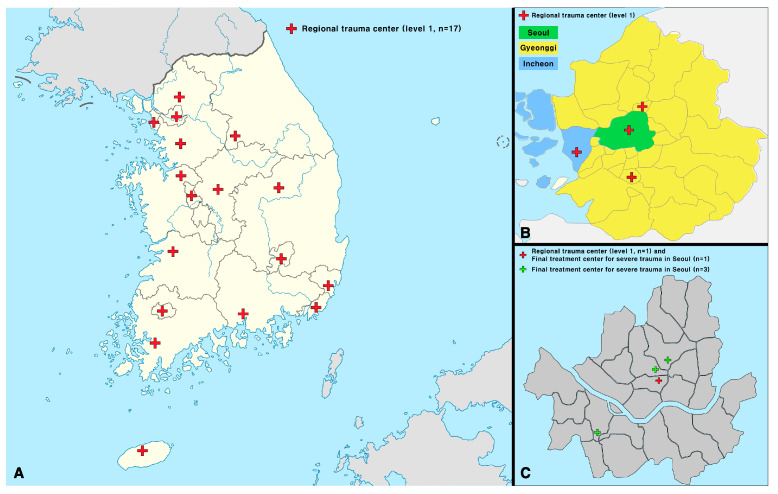
Locations and areas of the regional trauma centers in Seoul/Gyeonggi-Incheon and the Final Treatment Centers for Severe Trauma in Seoul. (**A**) The locations of the regional trauma centers in the Republic of Korea; (**B**) the locations of the regional trauma centers in Seoul and Gyeonggi-Incheon; (**C**) the locations of the regional trauma center and the Final Treatment Centers for Severe Trauma in Seoul. During the period from 2016 to 2020, when the data for this study were collected, the Seoul Regional Trauma Center had not yet opened. This figure was created using Adobe Illustrator 2023 (version 27.0, http://www.adobe.com/products/illustrator.html, [accessed on 5 January 2025]).

Even before the regional trauma center in Seoul opened in July 2023, the city had been operating four “Final Treatment Centers for Severe Trauma” since 2021. Three of the four centers were less equipped than the regional trauma centers. Among the tertiary hospitals, three were equipped with infrastructure to treat patients with trauma and functioned as Level II trauma centers. However, the outcomes of these centers have not been reported. The locations of the regional trauma centers in Seoul and Gyeonggi-Incheon and the Final Treatment Centers for Severe Trauma in Seoul are shown in Figure 3 [15,16].

### 2.5. The Insurance System in the Republic of Korea

Korean National Health Insurance (NHI) is a mandatory social insurance system. The MOHW outlines the health insurance policy, supervises its administration, and manages the system [17]. The Medical Aid Program (MAP) is a public assistance program designed to support low-income households by providing essential healthcare services, focusing on National Basic Livelihood Security Program recipients. MAP beneficiaries are categorized into two types based on factors such as age and employment capability. Type 1 beneficiaries included individuals under 18 or over 65 who could not work. Type 2 beneficiaries were adults aged 18–65 who could work [18]. The Republic of Korea’s Worker’s Compensation Insurance (WCI) is formally called Industrial Accident Compensation Insurance (IACI). Established under the 1963 IACI Act, this insurance system builds on the 1953 Labor Standards Act, which obligates employers to compensate employees for work-related injuries and illnesses [19].

### 2.6. Trauma Severity Definition

The ISS is an anatomical system used to assess the severity of trauma, with scores ranging from 1 to 75. To determine the ISS, the nine regions identified by the Abbreviated Injury Scale (AIS) were consolidated into six main categories: head or neck, face, chest, abdominal or pelvic contents, extremities or pelvic girdle, and external. The ISS was calculated by summing the squares of the highest AIS scores for the three most severely injured regions [20].

Since the 1980s, a score > 15 has generally indicated major trauma, which is associated with a mortality rate of >20% in these patients [21,22,23].

### 2.7. Statistical Analysis

The following variables were examined: sex, age, insurance type, ISS, hospital level, mechanism of injury, and in-hospital mortality (Table 1). Table 2 shows the time from 119 calls to Emergency Room (ER) visits, the number of surgeries performed, the time from ER visit to surgery, the number of angioembolizations performed, the time from ER visit to angioembolization, time from ER visit to first transfusion, and Glasgow Outcome Scale (GOS). All statistical analyses were conducted using the R software (version 4.3.1; R Foundation for Statistical Computing, Vienna, Austria) with 95% confidence intervals. The chi-square test was performed on nominal variables such as sex, insurance, hospital level, mechanism, and death. Student’s *t*-tests and Mann–Whitney U tests were performed based on the normality of continuous variables, including age, ISS, number of surgeries, angioembolizations, transfusions performed, time from 119 call to ER visit, time from ER visit to surgery, time from ER visit to the first transfusion, and GOS. Normality was evaluated by the Kolmogorov–Smirnov test. For the analysis of continuous variables, the data are presented as the mean ± standard deviation (SD) for normally distributed variables, and as the median with interquartile range (IQR) [Q1, Q3] for non-normally distributed variables.

Univariate and multivariable logistic regression analyses were performed (Table 3 and Table 4). Variables included in the multivariable logistic regression were those with *p* < 0.05, as determined by univariable logistic regression, and those considered clinically important. Variables included sex, age, insurance type, ISS, location, hospital level, mechanism, time from 119 calls to ER visits, number of surgeries performed, and number of angioembolizations performed.

The study was approved by the Institutional Review Board (IRB) of Korea University Anam Hospital (Approval No 2023AN0551), which waived the requirement for informed consent.

### 2.8. Reporting Guidelines

This study was conducted according to the Strengthening the Reporting of Observational Studies in Epidemiology guidelines to ensure the completeness and transparency of observational research.

## 3. Results

Patient characteristics according to the ISS group are summarized in Table 1. The proportion of male patients in the Gyeonggi-Incheon region was higher in the ISS < 9 and 9–15 groups. The mean age was consistently higher in Seoul across all groups. In terms of insurance type, the proportions individuals with of Motor Vehicle Insurance (MVI) and WCI increased with injury severity (MVI: 21.7%[*n* = 2451] vs. 22.5%[*n* = 846]/28.0%[*n* = 1014] vs. 34.1%[*n* = 494]/33.4%[*n* = 1002] vs. 38.7%[*n* = 514]; WCI: 0.4%[*n* = 43] vs. 2.2%[*n* = 84]/1.9%[*n* = 68] vs. 6.9%[*n* = 100]/3.1%[*n* = 94] vs. 9.7%[*n* = 129], respectively). The ISS (median) was significantly higher in Gyeonggi-Incheon in the ISS < 9 and ISS 9–15 groups (1[1, 4] vs. 2[1, 4], *p* < 0.001; 10[9, 12] vs. 10[9, 13], *p* < 0.001), although no statistically significant difference was observed in the ISS > 15 group (22[17, 26] vs. 22[17, 27], *p* = 0.287). Across hospital levels, the proportion of regional trauma centers in Seoul was lower in all three groups (0.2%[*n* = 26], 0.6%[*n* = 23], and 1.9%[*n* = 56], respectively). Conversely, in Gyeonggi-Incheon, the proportion of patients who visited regional trauma centers increased as injury severity increased (37.3%[*n* = 1404], 50.6%[*n* = 732], and 64.4%[*n* = 856]). In-hospital mortality rates after ER visits were not significantly different among the three groups (0.5%[*n* = 51] vs. 0.3%[*n* = 11], *p* = 0.241; 6.3%[*n* = 229] vs. 7.0%[*n* = 101], *p* = 0.434; 24.1%[*n* = 722] vs. 24.0%[*n* = 319], *p* = 0.986).

Table 2 presents the time required to visit the ER for 119 calls, time required for operation/angioembolization/first blood transfusion, number of surgeries, angioembolization, transfusions performed, and GOS results according to the ISS groups. The time from 119 calls to the ER visit (minutes, median) was significantly shorter in Seoul than in Gyeonggi-Incheon across all ISS groups (23[18, 29] vs. 24[18, 32], *p* < 0.001; 23[18, 30] vs. 25[18, 34], *p* < 0.001; 22[17, 28] vs. 25[18, 35], *p* < 0.001). The time from the ER visit to surgery (minutes, median) was significantly shorter in Gyeonggi-Incheon than in Seoul across all ISS groups (1522[482, 5712] vs. 1107[327, 5055.5], *p* = 0.001; 1733.5[454, 6362.8] vs. 1368[252.8, 6935.2], *p* = 0.002; 646[220, 7325] vs. 584[155.5, 6286.5], *p* < 0.001). The time from the ER visit to angioembolization (hours, median) was significantly shorter in Gyeonggi-Incheon than in Seoul in the ISS > 15 group (4.4[3.2, 6.6] vs. 2.8[1.9, 5.7], *p* = 0.001). The time from the ER visit to the first transfusion (minutes, median) was significantly shorter in Gyeonggi-Incheon than in Seoul across all ISS groups (113[60, 259] vs. 90.5[38.5, 194], *p* = 0.02; 172[82, 339.8] vs. 86[37.2, 206.2], *p* < 0.001; 130[67, 238] vs. 81[27, 165], *p* < 0.001). The rate of surgeries performed was higher in Gyeonggi-Incheon across all groups (*p* < 0.001). Additionally, the angioembolization rate was higher in Gyeonggi-Incheon in the ISS > 15 group (6.3%[*n* = 190] vs. 8.4%[*n* = 112]; *p* = 0.015). The transfusion rate was significantly higher in Seoul in the ISS > 15 group (41.7%[*n* = 1251] vs. 37.8%[*n* = 503], *p* = 0.018).

Table 3 and Table 4 show the results of the univariate and multivariable logistic regressions for risk factors related to in-hospital mortality of patients with trauma in Seoul and Gyeonggi-Incheon according to the ISS group.

Table 3 presents in-hospital mortality risk factors in Seoul according to the ISS group. The total number of patients was 17,911. The identified in-hospital mortality risk factors included **age** in all three groups (adjusted odds ratio [aOR] 1.08, 95% CI, 1.05–1.1, *p* < 0.001/aOR 1.04, 95% CI, 1.03–1.04, *p* < 0.001/aOR 1.03, 95% CI, 1.02–1.03, *p* < 0.001); regarding insurance type (reference = NHI), **NHI loss** in ISS 9–15 and ISS > 15 groups (aOR 2.89, 95% CI, 1.49–5.59, *p* = 0.002/aOR 2.52, 95% CI, 1.63–3.9, *p* < 0.001); **others** in ISS > 15 group (aOR 2.43, 95% CI, 1.37–4.32, *p* = 0.003); **ISS** in ISS > 15 group (aOR 1.08, 95% CI, 1.07–1.09, *p* < 0.001); regarding hospital level (reference = regional trauma centers), **regional emergency center** (aOR 2.39, 95% CI, 1.03–5.58, *p* = 0.043) and **local emergency center/institutes** (aOR 2.4, 95% CI, 1.04–5.53, *p* = 0.041) in ISS > 15 group; and **number of angioembolizations performed** in ISS < 9 and ISS 9–15 groups (aOR 14.07, 95% CI, 1.33–148.85, *p* = 0.028/aOR 5.07, 95% CI, 2.41–10.67, *p* < 0.001).

Table 4 presents the in-hospital mortality risk factors for Gyeonggi-Incheon according to the ISS. The total number of patients included was 6537. The identified in-hospital mortality risk factors included **age** in ISS 9–15 and ISS > 15 groups (aOR 1.04, 95% CI, 1.02–1.05, *p* < 0.001/aOR 1.02, 95% CI, 1.02–1.03, *p* < 0.001); and **ISS** in ISS < 9 and ISS > 15 groups (aOR 1.53, 95% CI, 1.15–2.03, *p* = 0.004/aOR 1.04, 95% CI, 1.02–1.05, *p* < 0.001); and, regarding the mechanism (reference = motor vehicle collisions), **falls and slippages** in ISS > 15 group (aOR 2.08, 95% CI, 1.38–3.14, *p* < 0.001); and **number of angioembolizations performed** in ISS 9–15 group (aOR 4.61, 95% CI, 1.61–13.23, *p* = 0.004).

## 4. Discussion

The population of Seoul is 9.5 million individuals, whereas that of New York City is 8.3 million individuals. New York City is approximately twice the size of Seoul in terms of geographical area and has 13 Level 1 trauma centers, 5 Level 2 trauma centers, and 3 Level 1 pediatric trauma centers [24]. Columbus City, Ohio, is comparable to Seoul in terms of geographical area but has a population of 950,000, two Level 1 trauma centers, two Level 2 trauma centers, and one Level 1 pediatric trauma center [25]. Trauma infrastructure and resources in Seoul are still insufficient compared to those in the United States.

As shown in Table 1, less than 2% of the patients in Seoul were directly transferred to regional trauma centers. The in-hospital mortality risk factors for the ISS > 15 group in Seoul were regional and local emergency centers or institutes (reference: regional trauma centers, aOR 2.39 and 2.4; Table 3). No regional trauma centers were opened in Seoul during the study period (2016–2020). The Seoul Regional Trauma Center was established in July 2023. Therefore, patients classified as being directly transferred to regional trauma centers in Seoul were transferred directly to regional trauma centers in Gyeonggi-Incheon, and most patients with trauma were directly transferred to regional emergency centers, local emergency centers, or institutes in Seoul. As shown in Table 4, the hospital level in Gyeonggi-Incheon was not identified as a risk factor for in-hospital mortality. This result suggests that the initial hospital selection based on patient severity is well implemented.

In Seoul, the time from a 119 call to the ER visit was shorter across all three groups compared to Gyeonggi-Incheon. This result may reflect the situation in Seoul, which had a large number of hospitals, but no regional trauma center, and used to transfer patients to the nearest hospital. In Gyeonggi-Incheon, the rates of surgeries performed were higher across all three groups, with a shorter time from an ER visit to surgery across all three groups, with a higher rate of angioembolization in the ISS > 15 group, and with a shorter time from an ER visit to angioembolization in the ISS > 15 group compared to Seoul (Table 2). These results may reflect the presence of three active regional trauma centers nationwide and the high severity of trauma in patients in these areas. The median times from ER visit to first blood transfusion for the ISS < 9, 9–15, and >15 groups were 90.5, 86, and 81 min, respectively, in Gyeonggi-Incheon, and 113, 172, and 130 min, respectively, in Seoul. The shorter transfusion time in Gyeonggi-Incheon may be associated with the well-established Massive Transfusion Protocol (MTP) system implemented in regional trauma centers. Furthermore, regional trauma centers consistently manage transfusion times as a quality control indicator [26]. The results of a study conducted as a sub-analysis of a multicenter, prospective, randomized controlled trial from the Pragmatic, Randomized Optimal Platelets and Plasma Ratios study revealed that from the time of MTP activation, with each minute of delay in administering blood products, the odds of mortality increased by 5% [27]. Therefore, medical institutions in Seoul should establish measures for rapid blood transfusions to reduce mortality rates.

As shown in Table 3 and Table 4, the unique in-hospital mortality risk factors in Seoul compared to those in Gyeonggi-Incheon were regional emergency centers, local emergency centers/institutes, NHI losses, and other insurances. Only 1.9% of patients with trauma in Seoul with an ISS > 15 were transferred directly to a regional trauma center. Most patients with trauma in Seoul are transferred directly to regional and local emergency centers or institutions that do not provide appropriate treatment. Meanwhile, patients transferred to regional trauma centers receive 24/7 care from dedicated trauma specialists, in facilities which are equipped with resuscitation rooms, intensive care units, operating rooms, and imaging laboratories (X-ray, computed tomography scan), exclusively for trauma patients.

People who cannot pay the NHI fee lose their eligibility for the NHI. Seoul has the highest population density in the Republic of Korea, and many impoverished individuals fall into social blind spots. The large population of Seoul has led to greater population diversity, which also contributes to other insurance as an in-hospital mortality risk factor.

To reduce the mortality of trauma patients in Seoul, concerted efforts should be directed toward mitigating these specific in-hospital mortality risk factors. Primarily because a regional trauma center is currently in operation in Seoul, patients with severe trauma must be directly transferred to this level 1 trauma center, bypassing regional and local emergency centers or institutes. Education at the prehospital level and collaboration with 119 Rescue are essential to more effectively enhance triage and select an initial transfer hospital for trauma centers.

The common in-hospital mortality risk factors for Seoul and Gyeonggi-Incheon were age, ISS, and angioembolization. The following is a comparison of the aOR of in-hospital mortality risk factors between Seoul and Gyeonggi-Incheon: age: 1.04 vs. 1.04 in the ISS 9–15 group; 1.03 vs. 1.02 in the ISS > 15 group; ISS: 1.08 vs. 1.04 in the ISS 9–15 group; angioembolization: 5.07 vs. 4.61 in the ISS 9–15 group. The aORs in Seoul were higher than those in Gyeonggi-Incheon for common in-hospital mortality risk factors between the two regions. This is thought to be due to the superior infrastructure provided by the regional trauma centers and the proper selection of transfer hospitals in Gyeonggi-Incheon, as well as the relatively poorer infrastructure for trauma care in Seoul during the study period.

Age is a well-known non-modifiable factor [28]. It is crucial to implement social measures to enhance safety to reduce the ISS. This could involve initiatives such as improving pedestrian passages to prevent pedestrian traffic accidents or implementing fall prevention projects to reduce falls and slippage, which are common injury mechanisms. Additionally, while currently abolished, projects that reduce vehicle speed limits in urban areas, such as the Seoul Downtown 5030 project, should be implemented to lower the traffic accident death rate and the overall number of accidents. According to the report, six months after implementation, the decrease in accidental pedestrian deaths in areas where the 5030 initiative was applied was 1.2 times greater than that on roads where the 5030 initiative was not introduced. Furthermore, compared to roads with a speed limit of 60 km/h under similar conditions, such as traffic volume, traffic accidents in areas where 5030 was implemented were reduced by 16.4–17.3% [29].

Angioembolization is considered one of the treatment options for patients with ongoing bleeding. While it is true that it is performed more frequently in higher-risk patients, this does not invalidate the findings of the study. If angioembolization were simply more common in patients with higher severity, the aOR would have decreased in the multivariable logistic regression analysis that included the ISS, which reflects the patients’ severity. However, the fact that the aOR increased when ISS was included suggests that angioembolization itself may be associated with increased in-hospital mortality. In the multivariable logistic regression analysis conducted separately for Seoul and Gyeonggi-Incheon, the inclusion of ISS further increased the aOR. In Seoul, the aOR increased from 4.86 to 5.07, and in Gyeonggi-Incheon, it increased from 3.77 to 4.61. These results suggest that angioembolization is associated with increased in-hospital mortality, even after adjusting for the severity of the patients through ISS. However, despite including ISS, the issue of reverse causality may still exist. While propensity score matching could be employed to address this potential bias, it is not appropriate for this study, as the study’s design focuses on investigating the regional characteristics rather than adjusting for confounding factors through matching.

Angioembolization was associated with in-hospital mortality risk in Seoul in the ISS < 9 and ISS 9–15 groups and in Gyeonggi-Incheon in the ISS 9–15 group. However, in Seoul, within the ISS < 9 group in Table 3, the aOR for angioembolization is 14.07. This value is very high, and the confidence interval is also wide (1.33–148.85). Upon reviewing the detailed data, we found 19 cases of angioembolization in the ISS < 9 group in Seoul (as shown in Table 2), with 1 mortality among them. Therefore, we conclude that these results should be interpreted with caution due to the very small sample size (*n* = 1). Further investigations into the use of angioembolization in ISS 9–15 groups in Seoul and Gyeonggi-Incheon are warranted.

The recently opened Seoul Regional Trauma Center has played a crucial role. Furthermore, it is necessary to assess outcomes following the opening of the Seoul Regional Trauma Center through follow-up research. Moreover, evaluating the outcomes of the Seoul Final Treatment Centers for Severe Trauma, which function as Level 2 trauma centers, is essential. Collaborative efforts should be undertaken with the Seoul Regional Trauma Center and Seoul Final Treatment Center for Severe Trauma to improve the outcomes of trauma patients in Seoul.

### Limitations

First, there is a potential selection bias arising from the study’s retrospective nature. However, as this study aimed to explore the differences between the two regions, it was unnecessary to employ statistical methods such as propensity score matching to mitigate confounding factors.

The second limitation relates to the characteristics of the databases used in this study. In Korea, the trauma registration system, known as the KTDB, is managed by the National Emergency Medical Center and is similar to the National Trauma Data Bank (NTDB) of the United States. The database used in this study lacked detailed clinical information on patients with trauma, such as vital signs, Glasgow Coma Scale score, and Revised Trauma Score. However, the KTDB is a hospital-based survey focusing exclusively on designated regional trauma centers. Consequently, post-transfer outcomes could not be verified, and patients outside regional trauma centers could not be traced. Moreover, it is difficult to compare these regions. However, unlike hospital-based surveys, Community-Based Severe Trauma Surveys provide only regional results. These surveys included information on the occurrence of trauma in response to 119 Rescue, ER visits, in-hospital treatment, and results after transfer. This database was revised and supplemented by consulting international trauma registries, such as the NTDB and Trauma Audit and Research Network, incorporating considerations for domestic data collection [30]. Consequently, this database has an advantage over the KTDB in identifying trauma patients in Seoul when regional trauma centers have not yet been established. The KTDB became publicly available in December 2023, paving the way for future research in this field.

## 5. Conclusions

The unique in-hospital mortality risk factors in Seoul, compared with those in Gyeonggi-Incheon, include regional emergency centers (ISS > 15), local emergency centers or institutes (ISS > 15), NHI loss (ISS 9–15 and ISS > 15), and the use of other insurance (ISS > 15). Collaborative efforts with the Seoul Regional Trauma Center and Seoul Final Treatment Centers for Severe Trauma are essential to reduce in-hospital mortality risk factors in Seoul’s trauma patients.

## Figures and Tables

**Table 1:** Patient characteristics according to the injury severity score (ISS) groups. Patients are divided into three ISS groups: ISS < 9 (mild trauma), ISS 9–15 (moderate trauma), ISS > 15 (severe trauma).

	ISS < 9			ISS 9–15			ISS > 15		
	Seoul	Gyeonggi-Incheon	*p*-Value	Seoul	Gyeonggi-Incheon	*p*-Value	Seoul	Gyeonggi-Incheon	*p*-Value
**Numbers**	11,296	3761		3617	1447		2998	1329	
**Sex**			0.042			0.003			0.172
Male	7437 (65.8)	2545 (67.7)		2560 (70.8)	1084 (74.9)		2218 (74.0)	1010 (76.0)	
Female	3859 (34.2)	1216 (32.3)		1057 (29.2)	363 (25.1)		780 (26.0)	319 (24.0)	
**Age** (mean)	44.0 ± 23.6	40.7 ± 23.1	<0.001	53.8 ± 20.9	50.7 ± 19.8	<0.001	53.1 ± 19.6	51.4 ± 18.6	0.006
**INSURANCE**			<0.001			<0.001			<0.001
NHI	7602 (67.3)	2438 (64.8)		2196 (60.7)	721 (49.8)		1592 (53.1)	573 (43.1)	
MVI	2451 (21.7)	846 (22.5)		1014 (28.0)	494 (34.1)		1002 (33.4)	514 (38.7)	
WCI	43 (0.4)	84 (2.2)		68 (1.9)	100 (6.9)		94 (3.1)	129 (9.7)	
MAP type 1	596 (5.3)	171 (4.5)		167 (4.6)	41 (2.8)		114 (3.8)	36 (2.7)	
MAP type 2	152 (1.3)	50 (1.3)		41 (1.1)	17 (1.2)		36 (1.2)	5 (0.4)	
NHI loss ^(a)^	300 (2.7)	163 (4.3)		85 (2.4)	71 (4.9)		103 (3.4)	68 (5.1)	
Others ^(b)^	152 (1.3)	9 (0.2)		46 (1.3)	3 (0.2)		57 (1.9)	4 (0.3)	
**ISS** (median) ^(c)^	1 [1, 4]	2 [1, 4]	<0.001	10 [9, 12]	10 [9, 13]	<0.001	22 [17, 26]	22 [17, 27]	0.287
**HOSPITAL LEVEL**			<0.001			<0.001			<0.001
Regional trauma centers	26 (0.2)	1404 (37.3)		23 (0.6)	732 (50.6)		56 (1.9)	856 (64.4)	
Regional emergency centers	2769 (24.5)	460 (12.2)		988 (27.3)	159 (11.0)		902 (30.1)	155 (11.7)	
Local emergency centers/institutes	8501 (75.3)	1897 (50.4)		2606 (72.0)	556 (38.4)		2040 (68.0)	318 (23.9)	
**MECHANISM OF INJURY**			<0.001			<0.001			<0.001
Motor vehicle collisions	3478 (30.8)	1125 (29.9)		1571 (43.4)	666 (46.0)		1478 (49.3)	736 (55.4)	
Falls and slippages	5914 (52.4)	1788 (47.5)		1818 (50.3)	631 (43.6)		1403 (46.8)	503 (37.8)	
Blunt injury	1113 (9.9)	465 (12.4)		112 (3.1)	69 (4.8)		64 (2.1)	52 (3.9)	
Penetrative injury	700 (6.2)	297 (7.9)		103 (2.8)	54 (3.7)		49 (1.6)	13 (1.0)	
Machine	91 (0.8)	86 (2.3)		13 (0.4)	27 (1.9)		4 (0.1)	25 (1.9)	
**Death** (after ER visit)	51 (0.5)	11 (0.3)	0.241	229 (6.3)	101 (7.0)	0.434	722 (24.1)	319 (24.0)	0.986

NHI, National Health Insurance; MVI, Motor Vehicle Insurance; WCI, Workers’ Compensation Insurance; MAP, Medical Aid Program; ISS, Injury Severity Score; ER, Emergency Room. ^(a)^ NHI disqualified person. ^(b)^ Police insurance, foreigner insurance, hull insurance, patriots, and veterans. ^(c)^ Data are presented as median [Q1, Q3], where Q1 and Q3 represent the first and third quartiles, respectively.

**Table 2:** Visiting time; number of surgeries, angioembolizations, and transfusions; time for surgeries, angioembolizations, and the first transfusion; and initial Glasgow outcome scale according to the injury severity score (ISS) groups. Patients are divided into three ISS groups: ISS < 9 (mild trauma), ISS 9–15 (moderate trauma), ISS > 15 (severe trauma).

	ISS < 9			ISS 9–15			ISS > 15		
	Seoul	Gyeonggi-Incheon	*p*-Value	Seoul	Gyeonggi-Incheon	*p*-Value	Seoul	Gyeonggi-Incheon	*p*-Value
**Numbers**	11,296	3761		3617	1447		2998	1329	
**Time from 119 call time to ER visit (min)**	23 [18, 29]	24 [18, 32]	<0.001	23 [18, 30]	25 [18, 34]	<0.001	22 [17, 28]	25 [18, 35]	<0.001
**Number of surgeries performed**	773 (6.8)	459 (12.2)	<0.001	1276 (35.3)	654 (45.2)	<0.001	1533 (51.1)	883 (66.4)	<0.001
**Time from the ER visit to surgery (min)**	1522 [482, 5712]	1107 [327, 5055.5]	0.001	1733.5 [454, 6362.8]	1368 [252.8, 6935.2]	0.002	646 [220, 7325]	584 [155.5, 6286.5]	<0.001
**Number of angio-embolizations performed**	19 (0.2)	5 (0.1)	0.815	54 (1.5)	29 (2.0)	0.241	190 (6.3)	112 (8.4)	0.015
**Time from the ER visit to angio-embolization (hr)**	12.7 [2.9, 131.9]	2.7 [2.3, 7.3]	0.189	4.4 [3.1, 7.0]	3.5 [2.9, 5.2]	0.252	4.4 [3.2, 6.6]	2.8 [1.9, 5.7]	0.001
**Number of transfusions performed**	228 (2.0)	96 (2.6)	0.059	539 (14.9)	228 (15.8)	0.47	1251 (41.7)	503 (37.8)	0.018
**Time from the ER visit to the first transfusion (min)**	113 [60, 259]	90.5 [38.5, 194]	0.02	172 [82, 339.8]	86 [37.2, 206.2]	<0.001	130 [67, 238]	81 [27, 165]	<0.001
**GOS initial**			0.296			0.332			0.156
Severe disability	27 (0.2)	4 (0.1)		18 (0.5)	3 (0.2)		11 (0.4)	1 (0.1)	
Moderate disability	141 (1.2)	46 (1.2)		85 (2.4)	32 (2.2)		29 (1.0)	17 (1.3)	
No disability	11,128 (98.5)	3711 (98.7)		3514 (97.2)	1412 (97.6)		2958 (98.7)	1311 (98.6)	

119, Korean emergency service call; ER, Emergency Room; GOS, Glasgow outcome scale. All time variable results are presented as medians (median [Q1, Q3]).

**Table 3:** Risk factors for in-hospital mortality in patients with trauma according to the ISS groups (Seoul). Patients are divided into three ISS groups: ISS < 9 (mild trauma), ISS 9–15 (moderate trauma), ISS > 15 (severe trauma).

Seoul (*n* = 17,911)	ISS < 9				ISS 9–15				ISS > 15			
	Univariable		Multivariable		Univariable		Multivariable		Univariable		Multivariable	
	Crude OR (95%CI)	Crude *p*-Value	Adjusted OR (95%CI)	Adjusted *p*-Value	Crude OR (95%CI)	Crude *p*-Value	Adjusted OR (95%CI)	Adjusted *p*-Value	Crude OR (95%CI)	Crude *p*-Value	Adjusted OR (95%CI)	Adjusted *p*-Value
**Sex (reference = Female)**	1.14 (0.65,2.02)	0.641			0.85 (0.63,1.15)	0.299			1.11 (0.92,1.34)	0.278		
**Age**	1.08 (1.06,1.1)	<0.001	1.08 (1.05,1.1)	<0.001	1.03 (1.02,1.04)	<0.001	1.04 (1.03,1.04)	<0.001	1.02 (1.02,1.03)	<0.001	1.03 (1.02,1.03)	<0.001
**Insurance (reference = NHI)**												
MVI	1.55 (0.83,2.89)	0.164			0.93 (0.68,1.27)	0.652			1.16 (0.96,1.39)	0.119		
WCI	0 (0,∞)	0.996			0.45 (0.11,1.85)	0.267			0.27 (0.12,0.59)	0.001	0.26 (0.12,0.59)	0.001
MAP type 1	2.57 (1.06,6.19)	0.036	1.19 (0.47,3)	0.714	0.74 (0.36,1.55)	0.429			0.81(0.5,1.3)	0.38		
MAP type 2	0 (0,∞)	0.992			0.37 (0.05,2.71)	0.328			0.81 (0.35,1.87)	0.629		
NHI loss ^(a)^	0 (0,∞)	0.988			2.67 (1.44,4.94)	0.002	2.89 (1.49,5.59)	0.002	2.32 (1.54,3.5)	<0.001	2.52 (1.63,3.9)	<0.001
Others ^(b)^	0 (0,∞)	0.992			2.22 (0.93,5.33)	0.074			2.45 (1.43,4.2)	0.001	2.43 (1.37,4.32)	0.003
**ISS**	1.09 (0.95,1.26)	0.212			0.96 (0.89,1.03)	0.275			1.07 (1.06,1.08)	<0.001	1.08 (1.07,1.09)	<0.001
**Hospital level (reference = regional trauma centers)**												
Regional emergency centers	0.15 (0.02,1.14)	0.066	0.26 (0.02,2.76)	0.263	432,305.86 (0,9.03)	0.966	403,412.26 (0,∞)	0.977	1.82 (0.85,3.91)	0.124	2.39 (1.03,5.58)	0.043
Local emergency centers/institutes	0.1 (0.01,0.76)	0.026	0.15 (0.01,1.58)	0.114	376,635.35 (0,7.87)	0.966	305,502.31 (0,∞)	0.977	1.97 (0.93,4.19)	0.079	2.4 (1.04,5.53)	0.041
**Mechanism (reference = Motor vehicle collisions)**												
Falls and slippages	0.93 (0.52,1.65)	0.8			1.19 (0.9,1.57)	0.215			1.1 (0.93,1.3)	0.273		
Blunt injury	0.16 (0.02,1.22)	0.078			0.73 (0.29,1.84)	0.511			0.53 (0.26,1.09)	0.085		
Penetrative injury	0.26 (0.03,1.95)	0.19			0.31 (0.08,1.28)	0.106			1.06 (0.55,2.05)	0.869		
Machine	0 (0,∞)	0.984			0 (0,∞)	0.975			0 (0,3.14)	0.963		
**Time from 119 call time to ER visit**	1 (0.98,1.01)	0.788			0.97 (0.95,0.98)	<0.001	0.96 (0.95,0.98)	<0.001	1 (1,1)	0.592		
**Number of surgeries performed**	0.85 (0.26,2.74)	0.786			0.68 (0.5,0.92)	0.011	0.73 (0.54,1)	0.05	0.62 (0.53,0.74)	<0.001	0.62 (0.52,0.75)	<0.001
**Number of angio-embolizations performed**	12.47 (1.63,95.25)	0.015	14.07 (1.33,148.85)	0.028	3.93 (2,7.72)	<0.001	5.07 (2.41,10.67)	<0.001	1.5 (1.09,2.06)	0.013	1.2 (0.84,1.71)	0.312

NHI, National Health Insurance; MVI, Motor Vehicle Insurance; WCI, Worker’s Compensation insurance; MAP, Medical Aid Program; ISS, Injury Severity Score; 119, Korean emergency service call; ER, Emergency Room. ^(a)^ NHI disqualified person. ^(b)^ Police insurance, foreigner insurance, hull insurance, patriots, and veterans.

**Table 4:** Risk factors for in-hospital mortality in patients with trauma according to the ISS groups (Gyeonggi-Incheon) Patients are divided into three ISS groups: ISS < 9 (mild trauma), ISS 9–15 (moderate trauma), ISS > 15 (severe trauma).

Gyeonggi-Incheon (*n* = 6537)	ISS < 9				ISS 9 –15				ISS > 15			
	Univariable		Multivariable		Univariable		Multivariable		Univariable		Multivariable	
	Crude OR (95%CI)	Crude *p*-Value	Adjusted OR (95%CI)	Adjusted *p*-Value	Crude OR (95%CI)	Crude *p*-Value	Adjusted OR (95%CI)	Adjusted *p*-Value	Crude OR (95%CI)	Crude *p*-Value	Adjusted OR (95%CI)	Adjusted *p*-Value
**Sex (reference = Female)**	1.75 (0.53,5.74)	0.358			1.35 (0.87,2.1)	0.179			1.4 (1.05,1.86)	0.021	1.27 (0.93,1.73)	0.126
**Age**	1.03 (1,1.06)	0.044	1.02 (0.99,1.05)	0.275	1.03 (1.02,1.05)	<0.001	1.04 (1.02,1.05)	<0.001	1.03 (1.02,1.03)	<0.001	1.02 (1.02,1.03)	<0.001
**Insurance (reference = NHI)**												
MVI	0 (0,∞)	0.992			1.14 (0.74,1.77)	0.552			0.76 (0.57,1.01)	0.055		
WCI	0 (0,∞)	0.997			0.57 (0.2,1.62)	0.292			0.71 (0.45,1.14)	0.155		
MAP type 1	1.59 (0.2,12.6)	0.662			0 (0,∞)	0.981			1.08 (0.51,2.3)	0.832		
MAP type 2	0 (0,∞)	0.998			0.86 (0.11,6.6)	0.882			0.7 (0.08,6.36)	0.755		
NHI loss ^(a)^	1.67 (0.21,13.23)	0.629			1.99 (0.93,4.24)	0.075			1.35 (0.79,2.32)	0.279		
Others ^(b)^	0 (0,∞)	0.999			0 (0,∞)	0.995			0.94 (0.1,9.11)	0.957		
**ISS**	1.32 (1.03,1.7)	0.03	1.53 (1.15,2.03)	0.004	0.87 (0.77,0.98)	0.021	0.86 (0.75,0.97)	0.02	1.03 (1.02,1.04)	<0.001	1.04 (1.02,1.05)	<0.001
**Hospital level (reference = Regional trauma centers)**												
Regional emergency centers	0.51 (0.06,4.23)	0.531	0.91 (0.1,8.21)	0.936	1.19 (0.62,2.3)	0.605	1.19 (0.6,2.38)	0.621	1.62 (1.11,2.36)	0.013	1.3 (0.86,1.94)	0.21
Local emergency centers/institutes	0.49 (0.14,1.75)	0.273	0.61 (0.16,2.35)	0.474	1.19 (0.77,1.83)	0.428	0.92 (0.58,1.46)	0.721	1.31 (0.98,1.77)	0.071	0.94 (0.67,1.32)	0.728
**Mechanism (reference = motor vehicle collisions)**												
Falls and slippages	24,817,221.73 (0,∞)	0.991			1.16 (0.77,1.76)	0.485			1.79 (1.38,2.32)	<0.001	2.08 (1.38,3.14)	<0.001
Blunt injury	13,608,211.79 (0,∞)	0.991			0.4 (0.1,1.69)	0.215			0.95 (0.46,1.93)	0.879		
Penetrative injury	64,430,717.0 (0,∞)	0.99			0.79 (0.24,2.64)	0.705			1.77 (0.54,5.81)	0.35		
Machine	1 (0,∞)	1			0 (0,∞)	0.976			0.17 (0.02,1.23)	0.079		
**Time from 119 call time to ER visit**	1 (0.99,1.01)	0.86			0.97 (0.95,0.99)	0.001	0.97 (0.96,0.99)	0.005	0.99 (0.98,0.99)	0.002	0.98 (0.97,0.99)	0.003
**Number of surgeries performed**	0.72 (0.09,5.62)	0.753			0.65 (0.43,0.99)	0.047	0.72 (0.46,1.12)	0.145	0.54 (0.41,0.7)	<0.001	0.52 (0.39,0.69)	<0.001
**Number of angio-embolizations performed**	0 (0,∞)	0.992			3.63 (1.44,9.14)	0.006	4.61 (1.61,13.23)	0.004	1.01 (0.64,1.58)	0.978		

NHI, National Health Insurance; MVI, Motor Vehicle Insurance; WCI, Worker’s Compensation insurance; MAP, Medical Aid Program; ISS, Injury Severity Score; 119, Korean emergency service call; ER, Emergency Room. ^(a)^ NHI disqualified person. ^(b)^ Police insurance, foreigner insurance, hull insurance, patriots, and veterans.

## Data Availability

The data analyzed in this study were obtained from the Korea Disease Control and Prevention Agency, and the following licenses and restrictions apply to the availability of these data. Requests to access these datasets should be directed to the official websites. (https://www.kdca.go.kr/injury/biz/injury/recsroom/rawDta/rawDtaDwldMain.do, (accessed on 5 January 2025)). However, the data are available from the authors upon reasonable request with permission from the Korea Disease Control and Prevention Agency (Point of contact: Hoonsung Park; first author; Email: niedwynn@naver.com).

## Supplementary Materials

The following supporting information can be downloaded at: https://www.mdpi.com/article/10.3390/jcm14051471/s1, Table S1. Definitions of preventability for death panel review.

## Abbreviations

The following abbreviations are used in this manuscript: PTDR, preventable trauma death rate; ISS, injury severity score; NHI, National Health Insurance; MOHW, Ministry of Health and Welfare; MAP, Medical Aid Program; WCI, Worker’s Compensation Insurance; IACI, Industrial Accident Compensation Insurance; AIS, Abbreviated Injury Scale; ER, Emergency Room; GOS, Glasgow Outcome Scale; MVI, Motor Vehicle Insurance; IRB, Institutional Review Board; MTP, Massive Transfusion Protocol; KTDB, Korea Trauma Data Bank; NTDB, National Trauma Data Bank.
