# Supplementary material for: Challenges in Saving Trauma Patients in Seoul Based on the 2016–2020 Community-Based Severe Trauma Survey
Source: J Clin Med. 2025 Feb 22;14(5):1471. doi: 10.3390/jcm14051471 (PMC11900097; doi:10.3390/jcm14051471)
Supplement: Supplementary file 1 [file jcm-14-01471-s001.zip › jcm-3472287-supplementary.pdf]

Table S1. Definitions of preventability for death panel review [3].

Preventability	Definitions
**A. Preventable**	• Injuries and sequelae considered survivable
	• Death could have been prevented if appropriate steps had been taken
	• Frank deviations from standard of care that, directly or indirectly, caused patient's death
	• Statistically, probability of survival greater than 50%, or ISS below 20
**B. Potentially preventable**	• Injuries and sequelae severe but survivable
	• Death potentially could have been prevented if appropriate steps had been taken
	• Evaluation and management generally appropriate
	• Some deviations from standard of care that may, directly or indirectly, have been implicated in patient's death
	• Statistically, probability of survival 25%–50% or ISS between 20 and 50
**C. Non-preventable**	• Injuries and sequelae non-survivable even with optimal management
	• Evaluation and management appropriate according to accepted standards
	• If patient had co-morbid factors, these were major contributors to death
	• Statistically, probability of survival less than 25% or ISS above 50
**D. Non-preventable, but with care that could have been improved**	• As with non-preventable above, but care is questionable or clear errors in care are detected, even though these did not lead to the death

ISS, Injury Severity Score
